# Multi-Constituent Simulation of Thrombus Deposition

**DOI:** 10.1038/srep42720

**Published:** 2017-02-20

**Authors:** Wei-Tao Wu, Megan A. Jamiolkowski, William R. Wagner, Nadine Aubry, Mehrdad Massoudi, James F. Antaki

**Affiliations:** 1 Department of Biomedical Engineering, Carnegie Mellon University, Pittsburgh, PA, 15213, USA; 2 McGowan Institute for Regenerative Medicine, Pittsburgh, PA, USA; 3 Department of Bioengineering, University of Pittsburgh, Pittsburgh, PA, USA; 4 Department of Surgery, University of Pittsburgh, Pittsburgh, PA, USA; 5 Department of Chemical Engineering, University of Pittsburgh, Pittsburgh, PA, USA; 6 Department of Mechanical Engineering, Northeastern University, Boston, MA, 02115, USA; 7 U. S. Department of Energy, National Energy Technology Laboratory (NETL), PA, 15236, USA

## Abstract

In this paper, we present a spatio-temporal mathematical model for simulating the formation and growth of a thrombus. Blood is treated as a multi-constituent mixture comprised of a linear fluid phase and a thrombus (solid) phase. The transport and reactions of 10 chemical and biological species are incorporated using a system of coupled convection-reaction-diffusion (CRD) equations to represent three processes in thrombus formation: initiation, propagation and stabilization. Computational fluid dynamic (CFD) simulations using the libraries of OpenFOAM were performed for two illustrative benchmark problems: *in vivo* thrombus growth in an injured blood vessel and *in vitro* thrombus deposition in micro-channels (1.5 mm × 1.6 mm × 0.1 mm) with small crevices (125 μm × 75 μm and 125 μm × 137 μm). For both problems, the simulated thrombus deposition agreed very well with experimental observations, both spatially and temporally. Based on the success with these two benchmark problems, which have very different flow conditions and biological environments, we believe that the current model will provide useful insight into the genesis of thrombosis in blood-wetted devices, and provide a tool for the design of less thrombogenic devices.

The hemostatic response at the site of vascular injury prevents the loss of blood, but excessive thrombosis may impede or interrupt blood flow to vital organs and tissues. The development of a thrombus in the vasculature is associated with myocardial infarction and stroke, as well as venous thromboembolic disorders^1^,^2^. Thrombus formation in blood-contacting medical devices is a common cause of failure, and one of the most significant sources of morbidity and mortality^3^,^4^. For instance, thrombosis in patients receiving ventricular assist devices (VADs) is one of the leading adverse events associated with this therapy, and has raised concerns in the medical community^5^,^6^,^7^,^8^ and with regulatory bodies such as the FDA^9^. Therefore, there is a critical need for improved understanding of the conditions under which hemostatic pathways may proceed to an excessive and undesirable thrombotic response.

Thrombosis is a complex phenomenon in which a combination of interrelated biochemical and hemodynamic factors result in several cascade reactions causing platelet activation, deposition, aggregation, and stabilization^10^,^11^,^12^. The complexity is accentuated by several feed-forward and feedback mechanisms promoting and inhibiting coagulation reactions. Therefore a comprehensive description of thrombus generation requires a model which can account for interrelated reactions involving platelet activation and aggregation, transport of platelets and chemical species in flow, and the interaction between the formed thrombus and the flow field^10^,^13^,^14^,^15^,^16^,^17^. The large number of chemical species and the complexity of cascade reactions make it very difficult to synthesize a comprehensive picture of coagulation dynamics using traditional laboratory approaches^16^. This motivates the pursuit of mathematical models^13^,^17^,^18^,^19^,^20^,^21^,^22^,^23^.

For the practical objective of predicting thrombosis in blood-wetted medical devices there is inevitably a tradeoff between complexity and utility. An overly simplistic model may fail to account for the essential mechanisms listed above. However, an overly complex model that includes the numerous biochemical species and pathways of coagulation may contain many unidentified parameters, and therefore be intractable or indeterminate to compute. A reasonable compromise was formulated by Sorensen *et al*. in which a set of convection-reaction-diffusion equations were employed to simulate platelet activation. This model featured a weighted linear combination of agonist concentrations, agonist release and synthesis by activated platelets, platelet-phospholipid-dependent generation of thrombin, and thrombin inhibition by heparin^13^,^14^. All of the parameters employed in Sorensen’s model were available from the experimental literature or by calibration with experimental data. However, this model has several shortcomings limiting its versatility, namely: (1) it limits platelet deposition to an idealized, unchanging surface-fluid boundary and does not account for alterations in the flow field due to thrombus growth, (2) it neglects shear-induced platelet activation, and (3) it does not include thrombus embolization (erosion) due to shear. These limitations motivated the development of a more sophisticated model that could account for these important mechanisms in the thrombotic process.

## Results

### Platelet deposition in blood vessel

**Figure 1:**
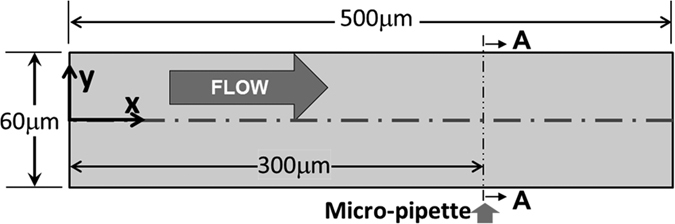
Schematic of the simulated blood vessel. The information about the dimensions of the blood vessel also can be found in Begent and Born^24^ and Born and Richardson^25^. Arrow (Micro-pipette) indicates location of ADP injection port (diameter = 3 *μm*). The surface properties were assumed to be those of healthy endothelium, with exception of the injection site at which there is an elevated rate of platelet adhesion.

The simulation of thrombus deposition in a blood vessel was modeled by assuming laminar flow in a straight, cylindrical blood vessel, shown in Fig. 1, that was defined to represent the seminal *in-vivo* experiments of Begent and Born^24^ and Born and Richardson^25^,^26^. In these experiments, thrombosis was initiated within a blood vessel of the hamster by injecting ADP at a localized injury site. The diameter and length of the simulated vessel was 0.06 mm and 0.5 mm, respectively. The mean velocity was 800 *μm*/*s* by prescribing a pressure difference between inlet and outlet, corresponding to a Reynold’s number of 0.14. The surface properties were assumed to be those of healthy endothelium, with the exception of the injured injection site, which was assumed to be highly adhesive to platelets. The corresponding reaction rates at the boundary were prescribed as *k*_*rpd,b*_ = 4.0 × 10^−5^ m/s and *k*_*apd,b*_ = 4.0 × 10^−4^ m/s, and the characteristic embolization shear rate was *τ*_*emb,b*_ = 1.0 *dyne* *cm*^−2^. (For the physical meaning of the terms/parameters, please see the tables in the methods section and supplemental documents.) The comparatively large value of *k*_*apd,b*_ = 4.0 × 10^−4^ m/s ensured that activated platelets nearby are efficiently captured by the injured site. The platelet deposition rate at the surface, *k*_*rpd,b*_was chosen based on the platelet-collagen deposition rate provided by Sorensen^13^,^14^. The platelet-platelet aggregation rates, *k*_*ra*_ = 3.0 × 10^−6^ m/s, *k*_*aa*_ = 3.0 × 10^−5^ m/s, were based on Sorensen *et al*. as well^13^,^14^. The characteristic embolization shear rate was *τ*_*emb*_ = 30 *dyne* *cm*^−2^ based on Goodman *et al*.^21^. The boundary reaction rates for normal, healthy endothelium were set to zero. All remaining parameters are provided in the tables in the methods section and supplemental documents. The inlet [RP] and [AP] were prescribed 6 × 10^14^ PLTs/m^3^ and 6 × 10^12^ PLTs/m^3^ respectively, based on normal values for rodents^27^,^28^. The [RP] and [AP] are the concentrations of unactivated (resting) platelets and activated platelets in flow respectively.

**Figure 2:**
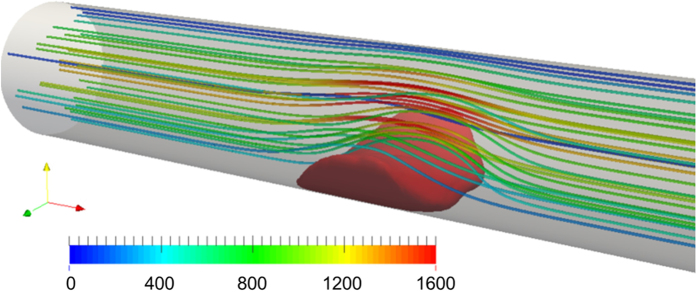
Snapshots of simulated thrombus and streamlines at t = 200 s with a mean velocity of 800 *μm/s*. The units of the color scale bar is *μm/s*.

**Figure 3:**
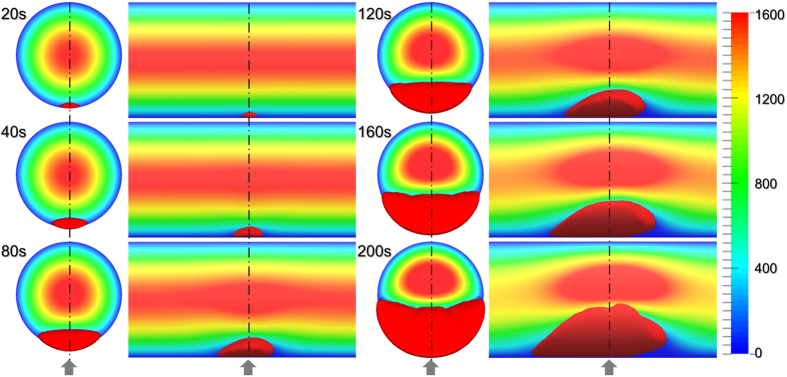
Orthographic views (front and side) of the progression of thrombus growth and velocity field evolution in a circular vessel at a mean velocity of 800 *μm/s*. The side view crosses the plane A-A shown in Fig. 1. The arrows indicate the position of the ADP injection port. The units of the scale bar is *μm/s*.

**Figure 4:**
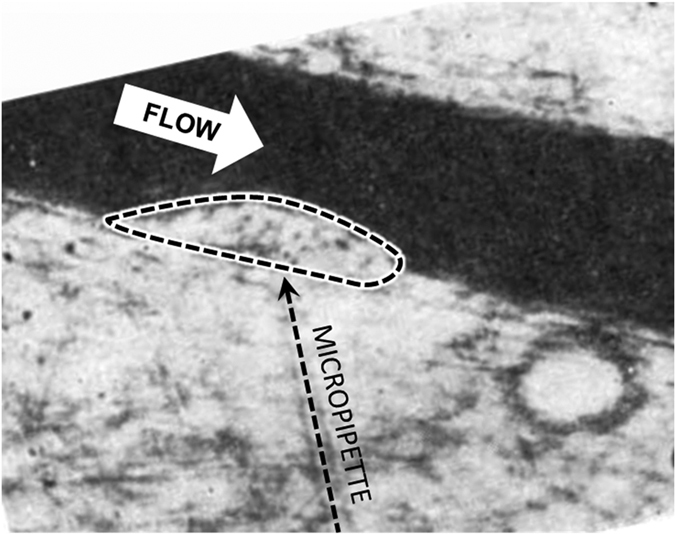
Thrombus in blood vessel observed by Begent and Born^24^ after approximately 100–200 s of ADP injection through micropipette. The figure is reprinted by permission from Macmillan Publishers Ltd: Nature. Begent and Born, Nature, 227, 926–930 (1970), copyright 1970^24^.

**Figure 5:**
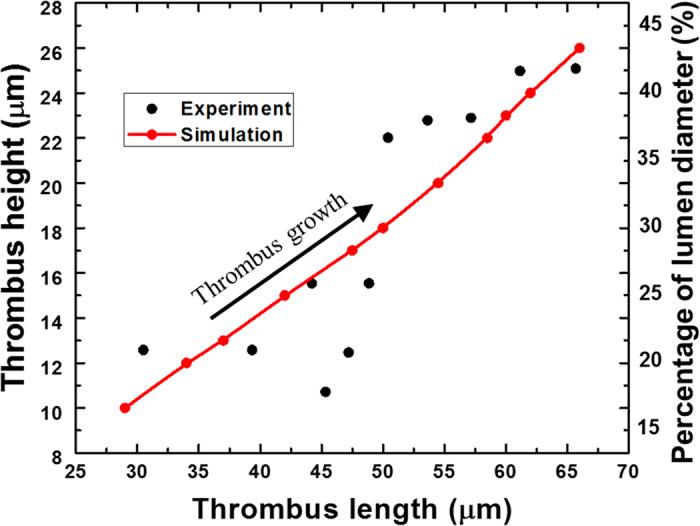
Thrombus height vs length by numerical simulation and experiments at a mean velocity of 800 *μm/s*. The experimental figure is reprinted by permission from Macmillan Publishers Ltd: Nature. Begent and Born, Nature, 227, 926–930 (1970), copyright 1970^24^. The experimental data points are taken from^26^ and replotted by the authors of the current paper.

Figure 2 provides a snapshot of the growing thrombus at t = 200 s. Comparison of the simulation results with experimental observations of Begent and Born^24^ were made on the basis of thrombus growth rate, shape, and size, (see Figs 3, 4 and 5). Figure 3 illustrates the progression of thrombus growth in the simulation. According to Begent and Born^24^, the thrombus grew to a height of 1/3 the lumen diameter in approximately 100–200 s, while the same size was obtained after about 150 s in our simulation. Figure 5 shows the comparison of the height versus the length of the thrombus observed experimentally and numerically. We found that the model accurately predicted the shape (aspect ratio) of the growing thrombus, which was approximately 2.5:1 (length:height) throughout its growth. The deviation between the experiments and simulations may be attributed to some level of uncertainty in the *in vivo* experiments. According to Begent and Born^24^,^25^,^26^, the data were derived from different experiments where the conditions, such as flow rate and vessel geometry, may have varied slightly. Qualitatively, it was observed that the thrombus grew both upstream and downstream of the injection site, which also agrees with the experimental observations. (See Fig. 4.) The upstream growth may be due to the capture of the incoming platelets and the relatively low shear rates; while downstream growth is supplied by platelets activated upstream.

**Figure 6:**
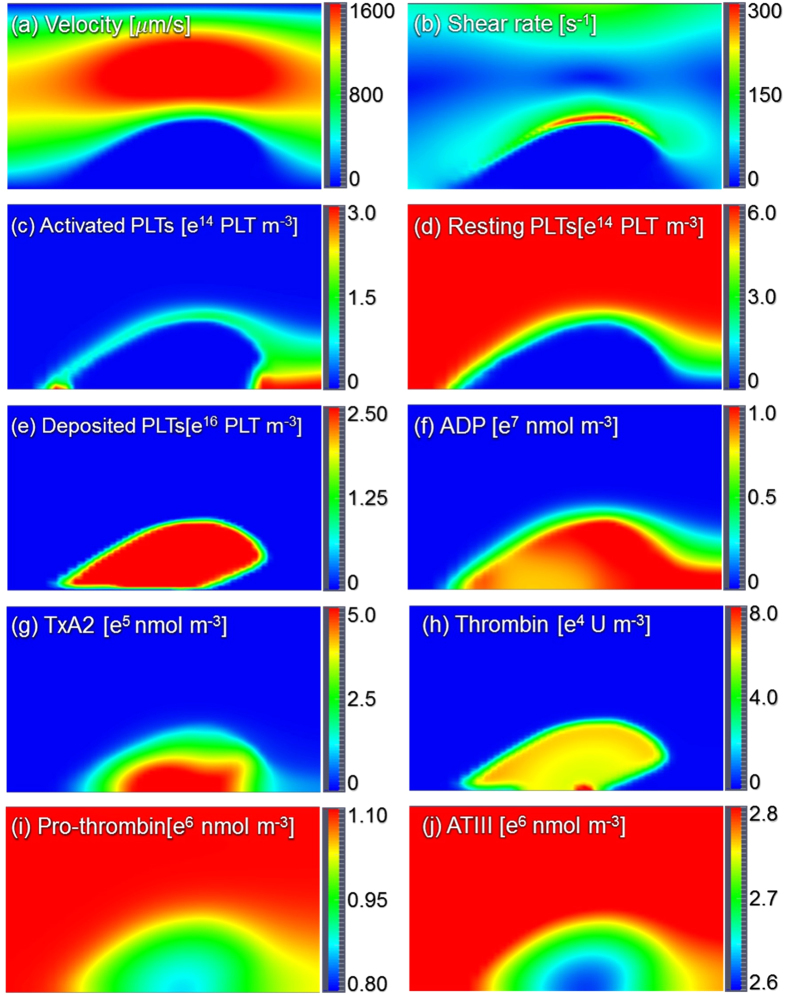
Streamlines, shear rate and concentration fields within the centerline plane at t = 200 s. It should be noted that the total activated platelets include the resting AP shown in figure (**c**) and deposited AP shown in figure (**e**).

**Figure 7:**
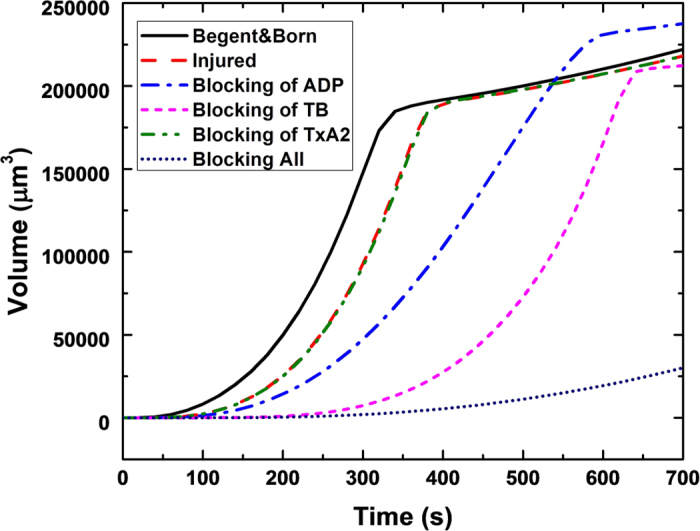
Time course of thrombus formation (volume) to total vessel occlusion with different agonists blocked. The plateaus exhibited by most of the cases correspond to occlusion of the vessel, followed by gradual longitudinal growth, driven primarily by diffusion.

Figure 6 shows the streamlines, shear rate, and concentration fields of the 8 remaining species on the radial-axial slice plane. The streamlines indicate that the fluid velocity within the region occupied by the thrombus is zero. Consequently, this produces a stenosis within the vessel. Figure 6(b) indicates a concentration of shear near the surface of the thrombus, which opposes platelet deposition. Figure 6(c) indicates that the concentration of activated platelets in the flow field is greatest at the leading and trailing edges of the thrombus. Figure 6(e) shows the concentration of the deposited activated platelets where we can find that the concentration in the thrombus core is comparatively higher, which is similar to the observation of Stalker *et al*.^29^. Figure 6(f) illustrates the diffusion of ADP from the injection site through the thrombus. The concentration of ADP on the surface activates resting platelets which causes the propagation of thrombus growth. Figure 6(g,h) indicates the synthesis of platelet agoinists, TxA_2_ and thrombin by deposited platelets in the interior of the thrombus. Accordingly Fig. 7(i,j) illustrates that prothrombin and anti-thrombin III (ATIII) are consumed in the interior of the thrombus.

Additional simulations were performed to study the rate, and time for complete occlusion of the vessel. Table 1 shows that blocking all platelet agonists highly suppresses thrombus growth, and blocking of ADP or Thrombin (TB) increases the occlusion time by almost a factor of 2. In contrast, the blocking of TxA_2_ has a negligible effect on occlusion time. Figure 7 provides the time course of platelet deposition, as measured by the total thrombus volume. We observed that the simulations predict a sigmoidal rate of deposition: initially slow, rapidly increasing until the vessel is completely occluded, at which point the thrombus continues to grow longitudinally at a slower rate. This pattern closely resembles the *in-vivo* observations of Mangin *et al*.^29^ in the blood vessel of a rat. An additional interesting observation from Fig. 7 is that for the case where ADP is blocked, the total thrombus volume at the time of occlusion is greater than the other cases. This implies that for this case, axial thrombus growth occurs more quickly than for the other cases.

### Platelet deposition in a micro-crevice

**Figure 8:**
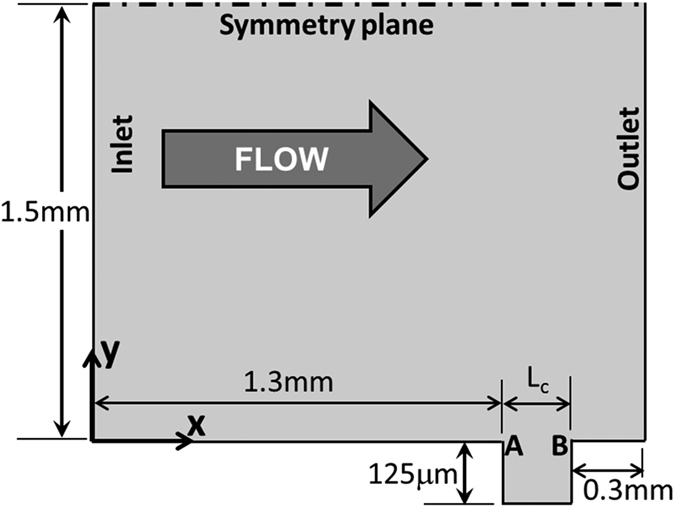
Schematic of the computational domain for rectangular microfluidic channel with a crevice. The overall half-height of the channel was 1.5 mm, and the depth is 0.1 mm. The domain only included a portion of the overall 3 cm length of the experimental channel. The height of the crevice was 0.125 mm and lengths, *L*_*c*_, of the crevices were 0.075 mm and 0.137 mm. The inlet velocity was 0.0173 m/s (Re = 32.30). A and B indicate upstream and downstream corners of the crevice, respectively.

The second benchmark problem, a rectangular channel with a micro crevice, was motivated by a persistent problem involved in most blood wetted medical devices, namely the seams and joints between component parts that are well known to be predisposed to thrombus deposition. The simulation was formulated to replicate a microfluidic experiment reported previously that utilized human blood^30^,^31^ (See Fig. 8). The half-height of the channel was 1.5 mm, and the depth was 0.1 mm. The modeled domain only included a portion of the overall 3 cm length of the experimental channel. The height of the crevice was 0.125 mm and the lengths of crevices studied here were *L*_*c*_ = 0.075 *mm* and *L*_*c*_ = 0.137 *mm*. The inlet velocity was prescribed as 0.0173 m/s, corresponding to a Reynolds number of 32.3. The reaction rates and characteristic embolization shear rates were the same as the previous simulation. However the boundary reaction rates and embolization rate needed to be adjusted to account for the different material properties (titanium alloy, Ti6Al4V): *k*_*rpdb*_ = 1.0 × 10^−20^ m/s, *k*_*apdb*_ = 1.0 × 10^−5^ m/s, *τ*_*embb*_ = 0.1 *dyne* *cm*^−2^. The value of *k*_*rpdb*_ implies no deposition occurs for resting unactivated platelets, and the values of *k*_*apdb*_ and *τ*_*embb*_ were chosen by best fitting the results of the case with *L*_*c*_ = 0.075 *mm*. The inlet [RP] and [AP] were prescribed as 2.5 × 10^14^ PLTs/m^3^ and 1 × 10^13^ PLTs/m^3^ respectively, corresponding to nominal values for human blood.

**Figure 9:**
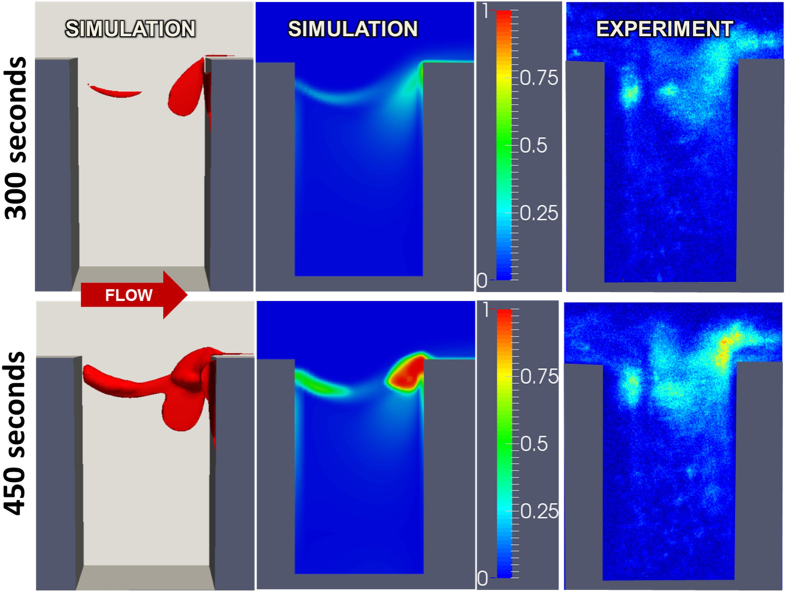
Comparison of thrombus deposition by simulation versus microscopic experiment at t = 300 and 450 s with crevice length *L*_*c*_ = 0.075 *mm*. Left panel is a 3D rendering of the simulated thrombus. Center and right panels indicate volume fraction of platelets in the near-wall region of the channel.

**Figure 10:**
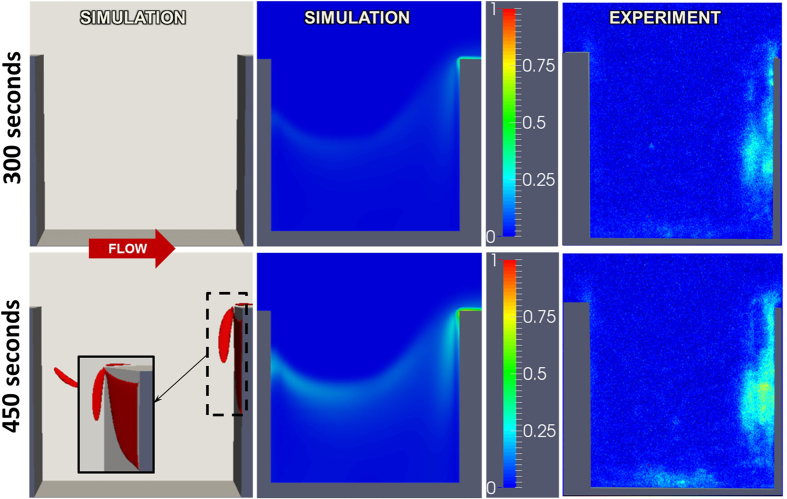
Comparison of thrombus deposition by simulation versus microscopic experiment at t = 300 and 450 s with crevice length *L*_*c*_ = 0.137 *mm*. Left panel is a 3D rendering of the simulated thrombus. Center and right panels indicate volume fraction of platelets in the near-wall region of the channel. The figures are reused by permission^31^.

Figure 9 shows the deposited platelet field based on the numerical simulation and experiments with the crevice length *L*_*c*_ = 0.075 *mm* at t = 300 s and 450 s. The first column is a three-dimensional rendering of the accumulated thrombus. The second column is the simulated volume fraction of platelets on the near wall, corresponding to the field of view of microscopic observations of fluorescently labelled platelets^32^,^33^. Comparison of simulation and experiment reveals very good agreement, particularly the thrombus growth at the leading and trailing edges of the crevice. Keeping all the conditions consistent, an additional simulation was performed in channel with a longer crevice (*L*_*c*_ = 0.137 *mm*.) Figure 10 shows the corresponding deposited platelets at t = 300 and 450 s. Compared to Fig. 9, it is clear that lengthening the crevice dramatically reduces the rate of deposition, which is consistent with experimental observations.

**Figure 11:**
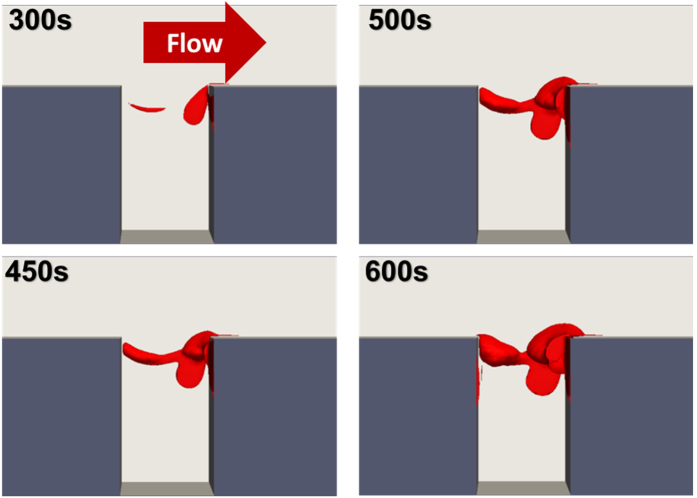
Evolution of thrombus growth in the rectangular crevice. The length of the crevice is 0.075 mm.

**Figure 12:**
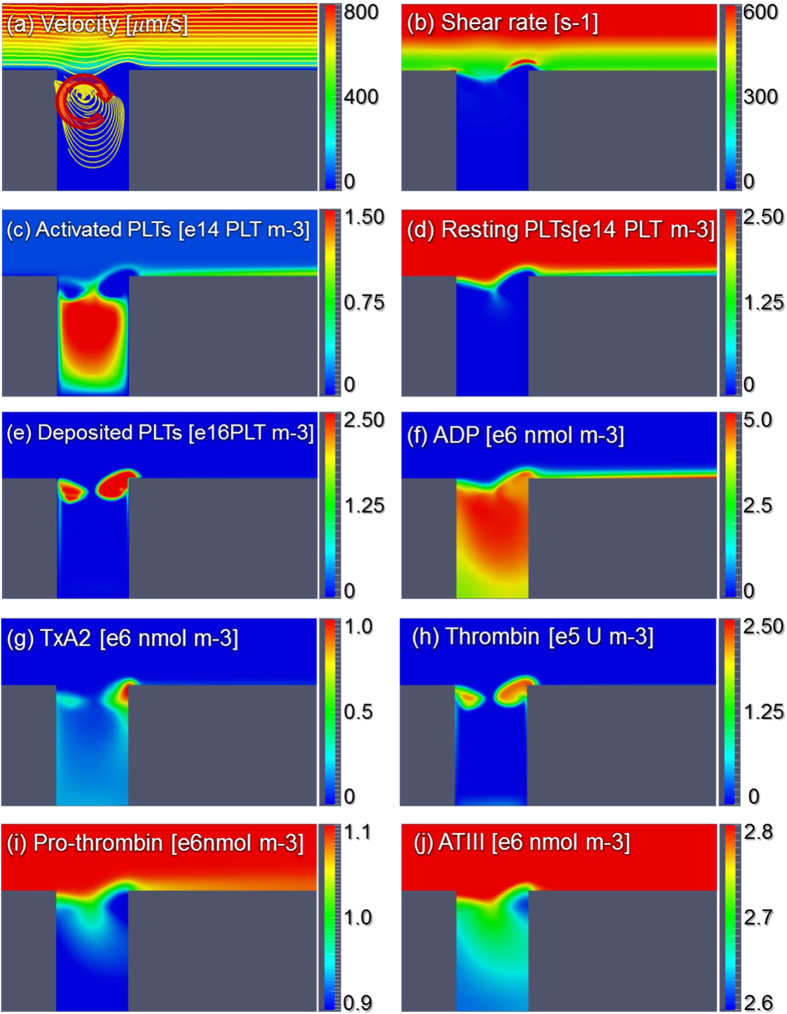
Streamlines, shear rate and concentration fields within the x-y slice plane at distance from the wall z = 10 *μm*, at t = 600 s. The length of the crevice is 0.075 mm.

Figure 11 shows the progression of the simulated thrombus deposition from 300–600 s illustrating the growth of both upstream and downstream thrombi with the crevice length of 0.075 mm. The downstream corner of the crevice has been indicated as the region where the thrombus grows earliest and fastest, (Fig. 11). This may be because at the rear corner, except for the platelets which were activated locally, many platelets are activated without being deposited at the leading corner. These platelets are transported to the rear corner where they are captured. This makes the growth rate of the thrombus at the rear corner much faster. When the deposited thrombus at the rear corner accumulates to a certain, rather large value, the agonists produced by those deposited platelets become concentrated enough to activate the unactivated surrounding platelets. In this region, the amount of the platelets activated by agonists will be much larger than those activated by the shear stress, which indicates an even faster thrombus deposition at the rear corner. Figure 12 provides the streamlines, shear rate and snapshot of concentration fields of the different species in the model. These figures indicate an accumulation of agonists and activated platelets within the cavity. Once thrombi begin to deposit at the leading and trailing edges, it appears that the secondary vortex within the cavity transports the activated platelets whereupon they accumulate with the deposited platelets in these two regions.

## Discussion

Mathematical models to simulate blood coagulation and thrombosis have steadily increased in complexity over recent decades^13^,^16^,^20^,^23^,^34^,^35^,^36^. Paradoxically, parallel efforts have been made to *reduce* the order, and hence complexity, of thrombosis models^37^,^38^. The appropriate level of complexity depends on the application for which the model is intended, and the questions being asked. For predicting thrombosis in blood wetted devices, the bare minimum that a model should include are the fundamental components of Virchow’s triad: (1) the properties of blood, including several states for platelets, biochemical agonists with associated positive feedback mechanisms, and agonist inhibition; (2) the character of flow which includes shear-mediated platelet activation, species convection, and thrombus-fluid interaction; and (3) surface chemistry in terms of surface reactivity.

Models commonly used today also encompass a wide range of physical scales and degree of granularity, each with advantages and disadvantages. In general, micro-scale models are more appropriate for studying phenomena that occur within and between individual cells^19^,^20^. Such models typically involve many unknown parameters. For this reason, phenomenological models are more practical, yet they are limited by the assumptions necessary to account for un-modeled behavior at the micro-scale. The thrombosis model presented here adopts a continuum approach that seeks a compromise in complexity and scale. The advantage of this approach is scalability, which enables simulation of arbitrarily shaped, complicated geometries that are encountered with the blood flow pathways of many current medical devices. To account for the most essential cell- and molecular scale mechanisms, this model employs a set of convection-diffusion-reaction equations that represent platelet activation by both chemical agonists and fluid forces; platelet deposition; thrombus propagation; thrombus embolization; thrombus stabilization; and inhibition. This model builds upon many of the principles incorporated in the earlier work of Sorensen *et al*.^13^,^14^, and inspired by the pioneering work of Fogelson^39^. Although the Sorensen model demonstrated good results in simulating platelet deposition in simple parallel-plate flow^13^,^14^, it lacked several features that limited its versatility. Accordingly, this revised model added shear-induced platelet activation, thrombus embolization due to shear, and fluid-solid interaction with the growing thrombus. The resulting number of species in the transport model was increased from 7 to 10. Similar to Sorensen, the current model derived most parameters from literature. The only parameters that were determined by experimental fitting were the three surface-specific coefficients, namely *k*_*rpd,b*_, *k*_*apd,b*_ and *τ*_*emb,b*_. If the model were to be used with different materials, such as polycarbonate or stainless steel, these three terms would need to be modified. Likewise, some parameters would need to be adjusted for viscosity, animal species, and various pathologies.

Although this model performed well with the two representative problems studied, it would still benefit from additional validation in different scenarios, such as vascular stenosis^40^,^41^, sudden expansions^33^, and other architectures with a broader range of flow conditions and blood properties^19^,^42^,^43^. It might be found that some assumptions and simplifications will need to be revisited. For example, we have modeled the blood as a single phase fluid, ignoring the spatial distribution of RBCs. This was done for the sake of numerical stability and in consideration of more complex future applications of the model. It is widely known that platelets tend to concentrate near the vessel wall, due to the interaction with RBCs, which can influence the rate of thrombosis^44^,^45^,^46^. The opposite is also possible: some experimental studies have also found that collisions of RBCs with thrombi reduced their mass^47^. These phenomena may have been compensated to some extent by choosing an appropriate deposition rates in the current work. Furthermore, the current model excluded the influence of von Willebrand factor, which is known to be relevant in platelet deposition in high-shear field devices such as blood pumps^48^,^49^,^50^,^51^. Also, the thrombus stabilization rate was assumed to be negligible in the current simulations. Consequently, thrombus growth was limited as deposition reached equilibrium with clearance by shear. However, for problems where deposition occurs over a long period of time, stabilization may play an essential role. Capturing this phenomenon is a challenge, since the process involves several chemical and biological species, such as fibrinogen/fibrin and factor XIII, which would increase the number of parameters^49^,^52^,^53^. Such an effort is a focal point for our ongoing work. The reader interested in fibrin formation and thrombus stabilization is referred to recent experimental reports by Colace, Neeves, and Savage^54^,^55^,^56^ and the theoretical model of Anand *et al*.^18^.

In its current embodiment, the thrombosis model presented here was able to replicate the patterns of platelet deposition in a micro-crevice observed experimentally. Both experiment and simulation revealed a counter-intuitive growth of thrombus from *both* edges (corners) of the crevice. The simulation however revealed the underlying processes by which an initial platelet deposition serves as a nidus for continued thrombus growth through the activation of resting platelets, generation of thrombin, and a temporal influence on the regional fluid dynamics. These results provide evidence that this model may find application in examining the influence of various hemodynamic and biochemical factors on the undesirable phenomenon of thrombotic deposition in medical devices.

## Conclusions

We have presented a mathematical model for simulating the initiation and propagation of thrombus formation on biomaterial surfaces. The model demonstrated excellent agreement with two illustrative benchmark problems: *in vivo* thrombus growth in an injured blood vessel and an *in vitro* thrombus deposition in micro-channels with small crevices. Because the model includes several interrelated biochemical and hemodynamic mechanisms, it was also able to simulate the influence of downregulating (blocking) certain biochemical agonists, such as ADP, thrombin, and TxA_2_. Using the model, we were able to predict the influence of a small crevice in the blood flow path, similar to those frequently found at the seams and joints between component parts of a blood-contacting medical device. By repeated simulations with a range of hemodynamic, biochemical, and geometric conditions, it is hoped that the model will prove useful as part of a broader design process seeking to create more blood biocompatible medical devices.

## Methods: Mathematical Model

We introduce a mathematical model of thrombosis consisting of equations of motion that determine the pressure and velocity fields and a set of coupled convection-diffusion-reaction (CDR) equations that govern the transport and inter-conversion of chemical and biological species–both within the thrombus and in the free stream. This model is developed based on the previous model of Sorensen *et al*.^13^,^14^. The changes and modifications are detailed in the Appendix.

### Equations of motion

Blood is treated as a multi-constituent mixture comprised of (1) a fluid phase which is modeled as a linear fluid and (2) a thrombus phase. The fluid phase comprises red blood cells (RBCs) suspended in plasma, and is governed by the following equations of conservation of mass and linear momentum:


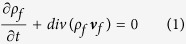



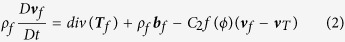


where ***T***_*f*_ is stress tensor of the fluid, represented by:


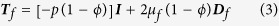


where *p* is the pressure, *μ*_*f*_ is the asymptotic dynamic viscosity (3.5 cP). A scalar field *ϕ* is introduced to represent the volume fraction of deposited platelets (thrombus). The density of the fluid phase is defined in terms of the volume fraction according to:


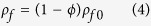


where *ρ*_*f*0_ is the density of the fluid phase (=1060 kg/m^3^), ***b***_*f*_ is the body force, ***v***_*f*_ and ***v***_*T*_ are the velocity of the fluid and thrombus phases, respectively. Therefore the term *C*_2_*  f(ϕ*)(***v***_*f*_ − ***v***_*T*_) is the resistance force on the fluid phase from the thrombus, where the coefficient *C*_2_ = 2 × 10^9^ *kg*/(*m*^3^*s*) is computed by assuming deposited platelets behave like densely compact particles 2.78 um in diameter, described by Johnson *et al*.^57^. and Wu *et al*.^58^,^59^, and *f(ϕ*) = *ϕ*(1 + 6.5*ϕ*) is the hindrance function.

### Convection-diffusion-reaction equations

**Figure 13:**
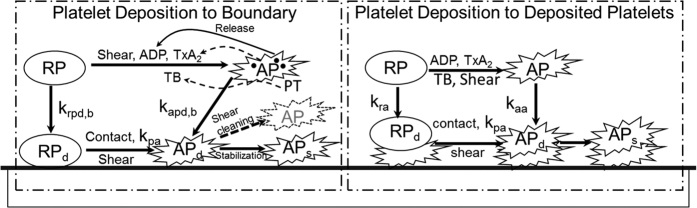
Schematic depiction of the thrombosis model, comprised of platelet deposition, aggregation, and stabilization. RP: resting platelet, AP: activated platelet, RP_d_ and AP_d_: deposited resting and active platelets, AP_s_: stabilized deposited active platelets. Agonists that cause activation (RP to AP) are adenosine diphosphate, ADP; thromboxane A_2_, TxA_2_; shear, and thrombin, TB–which is synthesized from prothrombin (PT). The suffix b refers to the reaction with the boundary (surface). The constants k_pa_, k_ra_, k_aa_, k_rpd,b_, k_apd,b_, refer to the reaction rates for inter-conversion of the associated platelet states.

The current model includes ten (10) chemical and biological species, summarized in Tables 2 and 3, and illustrated Fig. 13. These include five categories (states) of platelets (1) **RP**: resting platelets (in the flow field); (2) **AP**: activated platelets (in the flow field, and more reactive); (3) **RP**_**d**_: deposited (trapped) resting platelets, (4) **AP**_**d**_: deposited active platelets, and (5) **AP**_**s**_: deposited and stabilized platelets. An additional five biochemical species include (1) **a**_**pr**_: platelet-released agonists (ADP), (2) **a**_**ps:**_ platelet-synthesized agonist (thromboxane A_2_), which can be degraded via first-order reactions; (3) **PT:** prothrombin; (4) **TB:** thrombin, synthesized from prothrombin on the activated platelet phospholipid membrane; and (5) **AT:** anti-thrombin III, which inhibits thrombin and whose action is catalyzed by heparin via the kinetic model of Griffith. In the current model, value of most parameters are available from literatures.

Figure 13 depicts the fundamental mechanisms that comprise the thrombosis model, which include:

1. *Platelet Activation*: RP can be converted to be AP through exposure to shear^50^ and critical levels of biochemical agonists, including ADP^60^,^61^,^62^, Thrombin^63^,^64^ and TxA_2_^65^,^66^. It is assumed that this conversion results in the release of dense granules (ADP) and causes the platelets to become adherent to biomaterial surfaces and to other platelets.
2. *Platelet Deposition*: Both RP and AP in the free stream are able to deposit to a surface, such as a channel wall, whereupon they are designated RP_d_ and AP_d_, respectively. This process is mathematically modeled by a reaction flux at the boundary.
3. *Thrombus Propagation*: When the volume fraction of the deposited platelets (RP_d_ and AP_d_) is greater than a specified value, the deposition will propagate downstream. Additional details are provided in Supplemental Appendix.
4. *Thrombus Dissolution or Erosion*: Shear stress by the fluid phase is able to clear deposited platelets, thereby simulating the process known as surface “washing.” Mathematically, it was modeled by thrombus clearance term which is related to shear stress.
5. *Thrombus Stabilization*: Newly deposited platelets are converted by a constant rate to be as a solid clot which cannot be removed by hydrodynamic force.
6. *Thrombus Inhibition*: Anti-thrombin III in the free stream and within the thrombus may bind to thrombin, thereby neutralizing the effect of thrombin in activating additional platelets.
7. *Thrombus-fluid interaction*: The permeability of thrombus is inversely related to the volume fraction of deposited platelets. As represented in Equation (2) above, this is computed as a resistance force to fluid flow, analogous to the behavior of packed granular slurries.

The transport of the above species in the flow field is described by a corresponding set of CDR equations^13^,^14^ of the form:


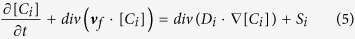


where [*C*_*i*_] is the concentration of species *i*; *D*_*i*_ refers to the diffusivity of species *i* in blood; and *S*_*i*_ is a reaction source term for species *i*. Deposited platelets (RP_d_, AP_d_, and AP_s_) are governed by corresponding rate equations:


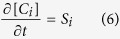


Tables 2 and 3 lists the appropriate form for the source terms *S*_*i*_, along with the abbreviations and the Table 4 shows the units, diffusion coefficients and normal level of concentrations for [*C*_*i*_].

Adhesion of platelets to surfaces and all the other species reactions at a boundary are modeled by surface-flux boundary conditions, following the approach of Sorensen *et al*.^13^,^14^. These are listed in Tables 5 and 6. The specific values and expressions of all the terms and their parameters are provided in the Tables 7 and 8. It should be mentioned that for different specific studies the only parameters that need to be determined are the three material surface-specific coefficients, namely *k*_*rpd,b*_, *k*_*apd,b*_ and *τ*_*emb,b*_. Here, similar to the reaction terms in the internal domain, a negative flux implies consumption and a positive flux implies generation.

The above equations were numerically simulated using the solvers and the libraries of OpenFOAM, a C++ toolbox^67^. The model was then evaluated for two benchmark problems: (1) growth of mural thrombus in an injured blood vessel and (2) deposition within a rectangular crevice. For each geometry studied, the domain was discretized as hexahedral meshes using ICEM. In each of the cases, mesh-dependence studies were performed to assure insensitivity to the mesh size. Numerical results were visualized with ParaView, a post-processing utility for the solution of continuum mechanics problems^68^.

## Additional Information

**How to cite this article**: Wu, W.-T. *et al*. Multi-Constituent Simulation of Thrombus Deposition. *Sci. Rep.* **7**, 42720; doi: 10.1038/srep42720 (2017).

**Publisher's note:** Springer Nature remains neutral with regard to jurisdictional claims in published maps and institutional affiliations.

## Supplementary Material

Supplementary Information

## Figures and Tables

**Table 1:** Time to vessel occlusion by thrombus under varying conditions.

Case	Occlusion time
ADP injected (same as Begent and Born^24^,^25^).	340 s
No ADP injected.	380 s
No ADP injected and with blocking of ADP.	590 s
No ADP injected and with blocking of TB.	620 s
No ADP injected and with blocking of TxA_2_.	380 s
No ADP injected and with blocking of all agonists.	N/A; (Up to 1200 s.)

For all of the cases there was an injured site as shown in Fig. 1, and all unmentioned conditions were the same as for the case studied previously^24^,^25^. The first case of Begent and Born implies no agonists were blocked, an injured site was present and ADP was injected at the injured site.

**Table 2:** Source terms associated with platelets.

Species	[*C*_*i*_] abbreviation	*S*_*i*_ form
Unactivated Resting PLTs	[RP]	−*k*_*apa*_[*RP*] − *k*_*spa*_[*RP*] − *k*_*rpa*_[*RP*]
Activated PLTs	[AP]	−*k*_*apa*_[*RP*] + *k*_*spa*_[*RP*] − *k*_*apa*_[*RP*]
Deposited Resting PLTs	[RP_d_]	(1 − *θ)k*_*rpd*_[*RP*] − *k*_*apa*_[*RP*_*d*_] − *k*_*spa*_[*RP*_*d*_] − *f*_*emb*_[*RP*_*d*_]
Deposited Activated PLTs	[AP_d_]	*θk*_*rpa*_[*RP*] + *k*_*apa*_[*AP*] + *k*_*apa*_[*RP*_*d*_] + *k*_*spa*_[*RP*_*d*_] − (*f*_*emb*_ + *f*_*stb*_)[*AP*_*d*_]
Deposited and stabilized PLTs	[AP_s_]	*f*_*stb*_[*AP*_*d*_]

See Fig. 13 for definition of reaction rates, k.

**Table 3:** Source terms for chemical species of the model.

Species	[*C*_*i*_] abbreviation	*S*_*i*_ form
PLT-released agonists (ADP)	[a_pr_]	*λ*_*j*_(*k*_*apa*_[*RP*] + *k*_*spa*_[*RP*] + *k*_*apa*_[*RP*_*d*_] + *k*_*spa*_[*RP*_*d*_] + *θk*_*rpd*_[*RP*]) − *k*_1_,_*j*_[a_pr_]
PLT-synthesized agonists (TxA_2_)	[a_ps_]	*s*_*pj*_([*AP*] + [*AP*_*d*_]) − *k*_1,*j*_ · [*a*_ps_]
Prothrombin	[PT]	−*ε*[PT](*ϕ*_*at*_([*AP*] + [*AP*_*d*_]) + *ϕ*_*rt*_([*RP*] + [*RP*_*d*_]))
Thrombin	[TB]	−Γ · [*TB*] + [*PT*](*ϕ*_*at*_([*AP*] + [*AP*_*d*_]) + *ϕ*_*rt*_([*RP*] + [*RP*_*d*_]))
ATIII	[AT]	−Γ · *ε*[*TB*]

**Table 4:** Species units, coefficient of species diffusion and initial condition.

Species	Species units	*D*_*i*_(*m*^2^*s*^−1^)	Initial (inlet) condition in blood
[RP]	PLT^−3^	1.58 × 10^−13^ + 6.0 × 10^−13^*γ*	1.5 × 10^14^ − 3.0 × 10^14^ (Human)
			1.9 × 10^14^ − 10.0 × 10^14^(Mouse)
[AP]	PLT^−3^	1.58 × 10^−13^ + 6.0 × 10^−13^*γ*	0.01[RP] − 0.05[Rp]
[a_pr_]	nmol m^−3^	2.57 × 10^−10^	0.0
[a_ps_]	nmol m^−3^	2.14 × 10^−10^	0
[PT]	nmol m^−3^	3.32 × 10^−11^	1.1 × 10^6^
[TB]	Um^−3^	4.16 × 10^−11^	0.0
[AT]	nmol m^−3^	3.49 × 10^−11^	2.844 × 10^6^
[RP_d_]	PLT^−3^	N/A	0.0
[AP_d_]	PLT^−3^	N	0.0
[AP_s_]	PLT^−3^	N/A	0.0

*γ* is the local shear rate. For more detail see Sorenson^13^,^14^ and Goodman^21^.

**Table 5:** Species boundary conditions.

Species [*C*_*i*_]	*j*_*i*_ form	Description
[RP]	−*Sk*_*rpdb*_[*RP*]	Consumption due to [RP]-surface adhesion; Generation due to shear embolization.
[AP]	−*Sk*_*apdb*_[*AP*]	Consumption due to [AP]-surface adhesion; Generation due to shear embolization.
[a_pr_]	*λ*_*j*_(*k*_*apa*_[*RP*_*d*_] + *k*_*spa*_[*RP*_*d*_] + *θ5k*_*rpdb*_[*RP*])	Generation due to agonists and shear activation of [RP_d_]; Generation due to surface contact activation of [RP]-surface adhesion.
[a_ps_]	*s*_*pj*_[*AP*_*d*_]	Platelet-synthesized generation due to [AP_d_]
[PT]	−*ε*[PT](*ϕ*_*at*_[*AP*_*d*_] + *ϕ*_*rt*_[*RP*_*d*_])	Consumption due to thrombin, [TB], generation.
[TB]	[PT](*ϕ*_*at*_[*AP*_*d*_] + *ϕ*_*rt*_[*RP*_*d*_])	Generation from prothrombin [PT] due to deposited platelets.
[AT]	0.0	No reaction flux.

**Table 6:** Species boundary conditions.

Species [*C*_*i*_]	*j*_*i*_ form	Description
[RP_d_]	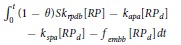	Generation due to [RP]-surface adhesion; Consumption due to agonists and shear activation; Consumption due to shear embolization.
[AP_d_]	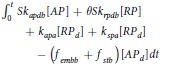	Generation due to [AP]-Surface adhesion; Generation due to surface contact activation of [RP]-Surface adhesion; Generation due to agonists and shear activation of [RP]; Consumption due to shear embolization and stabilization.
[AP_s_]	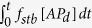	Generation due to stabilization.

(See Table 7 definitions of each of the terms).

**Table 7:** Value or expression and description of reaction terms and parameters in current paper.

Terms	Value or expression	units	Description
*k*_*apa*_	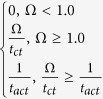	(s^−1^)	Platelets activation due to agonists; *t*_*ct*_ is the characteristic time, which can be used for adjusting the activation rate, and here we choose *t*_*ct*_ = 1*s* as provided Sorensen^13^,^14^; 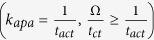 implies the reaction cannot be faster than platelets physical activation procedure and 99% platelets will be activated during the activation procedure if the agonists or shear stress is large enough *t*_*act*_ is the characteristic time; *t*_*act*_ is suggested to range from 0.1 s to 0.5 s considering the results by Frojmovic *et al*.^69^ and Richardson^70^.
Ω	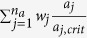	(N/A)	*a*_*j*_ refers to the concentration of ADP, TxA_2_ and Thrombin. Value of *w*_*j*_ and *a*_*j,crit*_ see Table 8
*k*_*spa*_	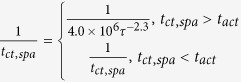	(s^−1^)	Platelet activation due to shear stress, *τ*. Expression *t*_*ct*,spa_ = 4.0 × 10^6^ *τ*^−2.3^ was provided by Goodman^21^ and Hellums^71^.
*k*_*rpd*_	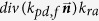	(s^−1^)	Unactivated platelets–deposited activated platelets ([RP]-[AP_d_]), deposition rate. *f* refers to the face of a mesh cell and 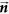 is the unit normal to the face. Details about how this term is calculated are provided in Supplemental Appendix.
*k*_*ra*_	3.0 × 10^−6^	(m s^−1^)	Constant related to *k*_*rpd*_^13^,^14^.
*k*_*apd*_	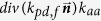	(s^−1^)	Activated platelets-deposited activated platelets ([AP]-[AP_d_]) deposition rate. Details about how this term is calculated are provided in Supplemental Appendix.
*k*_*aa*_	3.0 × 10^−5^	(m s^−1^)	Constant related to *k*_*apd*_^13^,^14^.
*f*_*emb*_	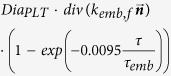	s^−1^	Platelet embolization due to shear stress; Expression *exp*(−0.0095*τ*) was suggested by Goodman^21^. *Dia*_*PLT*_ = 2.78 × 10^−6^ *m* is the hydraulic diameter of platelets.
*k*_*emb,f*_	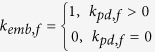	s^−1^	
*τ*_*emb*_	30	*dyne cm*^−2^	Platelet shear embolization related constant^21^.
*f*_*embb*_	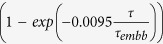	s^−1^	Platelet embolization in boundary due to shear stress.
*τ*_*embb*_	To be determined, dends on bio-material.	*dyne cm*^−2^	Patelet shear embolization related constant.
*PLT*_*max*_	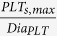	PLT m^−3^	The maximum concentration of platelets in space. *PLT*_*s,max*_ = 7 × 10^10^ *PLT*m^−2^ is the total capacity of the surface for platelets; *Dia*_*PLT*_ = 2.78 × 10^−6^ *m*.
*λ*_*j*_	2.4 × 10^−8^	mol PLT m^−3^	The amount of agonist *j* released per platelet.
*θ*	1.0	(N/A)	Platelets activation by contact.
*k*_1,*j*_	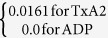	(s^−1^)	The inhibition rate constant of agonist.
*s*_*pi*_	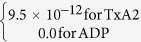	mol PLT^−3^ s	The rate constant of synthesis of an agonist.
*ε*	9.11 × 10^−3^	mol U^−1^	Unit conversion. From NIH units to SI units.
*ϕ*_*at*_	3.69 × 10^−15^	m^3^ nmol^−1^ PLT^−1^ Us^−1^	Thrombin generation rate on the surface of activated platelets.
*ϕ*_*rt*_	6.5 × 10^−16^	m^3^ nmol^−1^ PLT^−1^ Us^−1^	Thrombin generation rate on the surface of unactivated platelets.
Γ	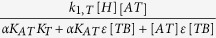	(s^−1^)	Griffith’s template model for the kinetics of the heparin-catalyzed inactivation of thrombin by ATIII.
*k*_1,*T*_	13.333	(s^−1^)	A first-order rate constant.
[*H*]	0.1 × 10^6^	nmol m^−3^	Heparin concentration, assuming specific activity of 300 U mg^−1^ and. molecular weight of 16 kDa^14^,^72^.
*α*	1.0	(N/A)	A factor to simulate a change in affinity of heparin for ATIII when it is bound to thrombin or for thrombin when it is bound to ATIII.
*K*_*AT*_	0.1 × 10^6^	nmol m^−3^	The dissociation constant for heparin/ATIII.
*K*_*T*_	3.50 × 10^4^	nmol m^−3^	The dissociation constant for heparin/thrombin.
*S*	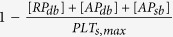	(N/A)	Pntage of the wall (boundary) not been occupied by deposited platelets.
*k*_*rpdb*_	To be determined, depends on bio-material.	(m s^−1^)	Unactivated platelet-boundary(wall) deposition rate.
*k*_*apdb*_	To be determined, depends on bio-material.	(m s^−1^)	Activated platelet-boundary(wall) deposition rate.
*f*_*stb*_	0.0	(s^−1^)	Deposited activated platelet stabilization rate.
*ϕ*	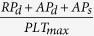	(N/A)	Volume fraction of deposited platelets.

**Table 8:** Threshold concentration of that agonist for platelet activation and agonist-specific weight^13^,^14^,^21^.

Species	*a*_*j,crit*_	*w*_*j*_
ADP([a_pr_])	1.00 × 10^6^ nmol m^−3^	1
TxA_2_([a_pr_])	1.20 × 10^6^ nmol m^−3^	3.3
Thrombin([TB])	0.1 × 10^6^ Um^−3^	30

It should be noticed that the threshold of the agonist may vary for some certain according to different experimental studies. For ADP see; refs 69, 73, 74, 75, 76 for Thrombin see; refs 77, 78, 79 for TxA_2_ see refs 80, 81, 82.

## References

[b1] Furie B. & Furie B. C. Mechanisms of thrombus formation. N. Engl. J. Med. 359, 938–949 (2008). 18753650 10.1056/NEJMra0801082

[b2] Handin R. I. Chapter 53: bleeding and thrombosis. Kasper D. L., Braunwald E., Fauci A. S. . Harrison’s Princ. Intern. Med. (16^th^ ed.). New York, NY: McGraw-Hill,. ISBN 71402357 (2005).

[b3] Jaffer I. H., Fredenburgh J. C., Hirsh J. & Weitz J. I. Medical device‐induced thrombosis: what causes it and how can we prevent it? J. Thromb. Haemost. 13, S72–S81 (2015). 26149053 10.1111/jth.12961

[b4] Reviakine I. . Stirred, shaken, or stagnant: What goes on at the blood–biomaterial interface. Blood Rev. (2016). 10.1016/j.blre.2016.07.003 27478147

[b5] Jeffries N. . What is the truth behind pump thrombosis in the HeartMate II device? A National Heart, Lung, and Blood Institute perspective based on data from the Interagency Registry for Mechanically Assisted Circulatory Support. J. Heart Lung Transplant. 34, 1505–10 (2015). 26681119 10.1016/j.healun.2015.10.036

[b6] Kirklin J. K. . Pump thrombosis in the Thoratec HeartMate II device: An update analysis of the INTERMACS Registry. J. Heart Lung Transplant. 34, 1515–26 (2015). 26681121 10.1016/j.healun.2015.10.024

[b7] Blitz A. Pump thrombosis-A riddle wrapped in a mystery inside an enigma. Ann. Cardiothorac. Surg. 3, 450–71 (2014). 25452905 10.3978/j.issn.2225-319X.2014.09.10 PMC4229470

[b8] Najjar S. S. . An analysis of pump thrombus events in patients in the HeartWare ADVANCE bridge to transplant and continued access protocol trial. J. Hear. Lung Transplant. 33, 23–34 (2014). 10.1016/j.healun.2013.12.001 24418731

[b9] Cipriano M. HeartWare Pauses Enrollment in Clinical Trial of MVAD Heart Pump | FDANews. Available at: http://www.fdanews.com/articles/173075-heartware-pauses-enrollment-in-clinical-trial-of-mvad-heart-pump. (Accessed: 11^th^ September 2015) (2015).

[b10] Skorczewski T., Erickson L. C. & Fogelson A. L. Platelet motion near a vessel wall or thrombus surface in two-dimensional whole blood simulations. Biophys J 104, 1764–1772 (2013). 23601323 10.1016/j.bpj.2013.01.061 PMC3628562

[b11] Lu Y. . Inhibitory effect of caffeic acid on ADP-induced thrombus formation and platelet activation involves mitogen-activated protein kinases. Sci. Rep. 5 (2015). 10.1038/srep13824 PMC4561902 26345207

[b12] Liang M.-L. . Pentamethylquercetin (PMQ) reduces thrombus formation by inhibiting platelet function. Sci. Rep. 5 (2015). 10.1038/srep11142 PMC4461919 26059557

[b13] Sorensen E. N., Burgreen G. W., Wagner W. R. & Antaki J. F. Computational simulation of platelet deposition and activation: I. Model development and properties. Ann Biomed Eng 27, 436–448 (1999). 10468228 10.1114/1.200

[b14] Sorensen E. N., Burgreen G. W., Wagner W. R. & Antaki J. F. Computational simulation of platelet deposition and activation: II. Results for Poiseuille flow over collagen. Ann Biomed Eng 27, 449–458 (1999). 10468229 10.1114/1.201

[b15] Fogelson A. L., Hussain Y. H. & Leiderman K. Blood clot formation under flow: the importance of factor XI depends strongly on platelet count. Biophys J 102, 10–18 (2012). 22225793 10.1016/j.bpj.2011.10.048 PMC3250692

[b16] Kuharsky A. L. & Fogelson A. L. Surface-mediated control of blood coagulation: the role of binding site densities and platelet deposition. Biophys J 80, 1050–1074 (2001). 11222273 10.1016/S0006-3495(01)76085-7 PMC1301304

[b17] Ataullakhanov F. I. & Panteleev M. A. Mathematical modeling and computer simulation in blood coagulation. Pathophysiol. Haemost. Thromb. 34, 60–70 (2006). 10.1159/000089927 16432308

[b18] Anand M., Rajagopal K. & Rajagopal K. R. A model for the formation and lysis of blood clots. Pathophysiol. Haemost. Thromb. 34, 109–120 (2006). 10.1159/000089931 16432312

[b19] Flamm M. H. . Multiscale prediction of patient-specific platelet function under flow. Blood 120, 190–198 (2012). 22517902 10.1182/blood-2011-10-388140 PMC3390957

[b20] Xu Z. . A multiscale model of venous thrombus formation with surface-mediated control of blood coagulation cascade. Biophys J 98, 1723–1732 (2010). 20441735 10.1016/j.bpj.2009.12.4331 PMC2862154

[b21] Goodman P. D., Barlow E. T., Crapo P. M., Mohammad S. F. & Solen K. A. Computational model of device-induced thrombosis and thromboembolism. Ann. Biomed. Eng. 33, 780–797 (2005). 16078618 10.1007/s10439-005-2951-z

[b22] Anand M., Rajagopal K. & Rajagopal K. R. A model incorporating some of the mechanical and biochemical factors underlying clot formation and dissolution in flowing blood: review article. J. Theor. Med. 5, 183–218 (2003).

[b23] Xu Z., Chen N., Kamocka M. M., Rosen E. D. & Alber M. A multiscale model of thrombus development. J. R. Soc. Interface 5, 705–722 (2008). 17925274 10.1098/rsif.2007.1202 PMC2607450

[b24] Begent N. & Born G. V. R. Growth rate *in vivo* of platelet thrombi, produced by iontophoresis of ADP, as a function of mean blood flow velocity. Nature 227, 926–930 (1970). 5448995 10.1038/227926a0

[b25] Born G. V. R. & Richardson P. D. Activation time of blood platelets. J. Membr. Biol. 57, 87–90 (1980). 7009877 10.1007/BF01868994

[b26] Begent N. A. Loealised reactions of the microcirculation of the hamster cheek pouch to substances administered by iontophoresis, with particular reference to intravascular platelet aggregation (University of London, 1971).

[b27] Harrison J. . Additive effect of erythropoietin and heme on murine hematopoietic recovery after azidothymidine treatment [see comments]. Blood 82, 3574–3579 (1993). 8260696

[b28] Corash L., Mok Y., Levin J. & Baker G. Regulation of platelet heterogeneity: effects of thrombocytopenia on platelet volume and density. Exp. Hematol. 18, 205–212 (1990). 2303115

[b29] Stalker T. J. . A systems approach to hemostasis: 3. Thrombus consolidation regulates intrathrombus solute transport and local thrombin activity. Blood blood 2014-01-550319 (2014). 10.1182/blood-2014-01-550319 PMC4162112 24951426

[b30] Jamiolkowski M. A., Woolley J. R., Kameneva M. V., Antaki J. F. & Wagner W. R. Real time visualization and characterization of platelet deposition under flow onto clinically relevant opaque surfaces. J. Biomed. Mater. Res. - Part A 103, 1303–1311 (2015). 10.1002/jbm.a.35202 PMC4203704 24753320

[b31] Jamiolkowski M. A., Pedersen D. D., Wu W.-T., Antaki J. F. & Wagner W. R. Visualization and analysis of biomaterial-centered thrombus formation within a defined crevice under flow. Biomaterials 96, 72–83 (2016). 27156141 10.1016/j.biomaterials.2016.04.022 PMC4982661

[b32] Wu W.-T., Yang F., Antaki J. F., Aubry N. & Massoudi M. Study of blood flow in several benchmark micro-channels using a two-fluid approach. Int. J. Eng. Sci. 95, 49–59 (2015). 26240438 10.1016/j.ijengsci.2015.06.004 PMC4521229

[b33] Zhao R. . Micro-flow visualization of red blood cell-enhanced platelet concentration at sudden expansion. Ann. Biomed. Eng. 36, 1130–1141 (2008). 18418710 10.1007/s10439-008-9494-z PMC6390961

[b34] Voronov R. S., Stalker T. J., Brass L. F. & Diamond S. L. Simulation of intrathrombus fluid and solute transport using *in vivo* clot structures with single platelet resolution. Ann. Biomed. Eng. 41, 1297–1307 (2013). 23423707 10.1007/s10439-013-0764-z PMC3640668

[b35] Anand M., Rajagopal K. & Rajagopal K. R. A model for the formation, growth, and lysis of clots in quiescent plasma. A comparison between the effects of antithrombin III deficiency and protein C deficiency. J. Theor. Biol. 253, 725–738 (2008). 18539301 10.1016/j.jtbi.2008.04.015

[b36] Zhu D. Mathematical modeling of blood coagulation cascade: kinetics of intrinsic and extrinsic pathways in normal and deficient conditions. Blood Coagul. Fibrinolysis 18, 637–646 (2007). 17890951 10.1097/MBC.0b013e3282a167bb

[b37] Mehrabadi M., Casa L. D. C., Aidun C. K. & Ku D. N. A Predictive Model of High Shear Thrombus Growth. Ann. Biomed. Eng. (2016). 10.1007/s10439-016-1550-5 26795978

[b38] Taylor J. O., Meyer R. S., Deutsch S. & Manning K. B. Development of a computational model for macroscopic predictions of device-induced thrombosis. Biomech. Model. Mechanobiol. 1–19 (2016). 10.1007/s10237-016-0793-2 27169403

[b39] Fogelson A. L. Continuum models of platelet aggregation: formulation and mechanical properties. SIAM J. Appl. Math. 52, 1089–1110 (1992).

[b40] Para A. N. & Ku D. N. A low-volume, single pass *in-vitro* system of high shear thrombosis in a stenosis. Thromb. Res. 131, 418–424 (2013). 23535566 10.1016/j.thromres.2013.02.018

[b41] Bark D. L. & Ku D. N. Platelet transport rates and binding kinetics at high shear over a thrombus. Biophys J 105, 502–511 (2013). 23870271 10.1016/j.bpj.2013.05.049 PMC3714887

[b42] Merino A., Cohen M., Badimon J. J., Fuster V. & Badimon L. Synergistic action of severe wall injury and shear forces on thrombus formation in arterial stenosis: definition of a thrombotic shear rate threshold. J. Am. Coll. Cardiol. 24, 1091–1097 (1994). 7930203 10.1016/0735-1097(94)90875-3

[b43] Badimon L., Badimon J. J., Turitto V. T. & Fuster V. Thrombosis: Studies under flow conditions. Ann. N. Y. Acad. Sci. 516, 427–540 (1987). 3439747

[b44] Turitto V. T. & Weiss H. J. Red blood cells: their dual role in thrombus formation. Science 207, 541–543 (1980). 7352265 10.1126/science.7352265

[b45] Vahidkhah K., Diamond S. L. & Bagchi P. Platelet dynamics in three-dimensional simulation of whole blood. Biophys. J. 106, 2529–2540 (2014). 24896133 10.1016/j.bpj.2014.04.028 PMC4052243

[b46] Crowl L. M. & Fogelson A. L. Computational model of whole blood exhibiting lateral platelet motion induced by red blood cells. Int. j. numer. method. biomed. eng. 26, 471–487 (2010). 21152372 10.1002/cnm.1274 PMC2997713

[b47] Chesnutt J. K. W. & Han H.-C. Effect of red blood cells on platelet activation and thrombus formation in tortuous arterioles. Front. Bioeng. Biotechnol. 1 (2013). 10.3389/fbioe.2013.00018 PMC4090894 25022613

[b48] Colace T. V. & Diamond S. L. Direct observation of von Willebrand factor elongation and fiber formation on collagen during acute whole blood exposure to pathological flow. Arterioscler. Thromb. Vasc. Biol. 33, 105–113 (2013). 23104847 10.1161/ATVBAHA.112.300522 PMC3595169

[b49] Ruggeri Z. M. & Jackson S. P. Platelet thrombus formation in flowing blood. Platelets 399 (2013).24047408

[b50] Herbig B. A. & Diamond S. L. Pathological von Willebrand factor fibers resist tissue plasminogen activator and ADAMTS13 while promoting the contact pathway and shear‐induced platelet activation. J. Thromb. Haemost. 13, 1699–1708 (2015). 26178390 10.1111/jth.13044 PMC4560981

[b51] Zlobina K. E. & Guria G. T. Platelet activation risk index as a prognostic thrombosis indicator. Sci. Rep. 6 (2016). 10.1038/srep30508 PMC4962318 27461235

[b52] Jackson S. P., Nesbitt W. S. & Kulkarni S. Signaling events underlying thrombus formation. J. Thromb. Haemost. 1, 1602–1612 (2003). 12871297 10.1046/j.1538-7836.2003.00267.x

[b53] Zubairova L. D. . Circulating Microparticles Alter Formation, Structure, and Properties of Fibrin Clots. Sci. Rep. 5 (2015). 10.1038/srep17611 PMC4669434 26635081

[b54] Colace T. V., Muthard R. W. & Diamond S. L. Thrombus growth and embolism on tissue factor-bearing collagen surfaces under flow role of thrombin with and without fibrin. Arterioscler. Thromb. Vasc. Biol. 32, 1466–1476 (2012). 22516070 10.1161/ATVBAHA.112.249789 PMC3418805

[b55] Neeves K. B., Illing D. A. R. & Diamond S. L. Thrombin flux and wall shear rate regulate fibrin fiber deposition state during polymerization under flow. Biophys. J. 98, 1344–1352 (2010). 20371335 10.1016/j.bpj.2009.12.4275 PMC2849060

[b56] Savage B. & Ruggeri Z. M. Platelet thrombus formation in flowing blood. Platelets, 359–367 (2007).

[b57] Johnson G., Massoudi M. & Rajagopal K. R. Flow of a fluid—solid mixture between flat plates. Chem. Eng. Sci. 46, 1713–1723 (1991).

[b58] Wu W.-T., Aubry N. & Massoudi M. On the coefficients of the interaction forces in a two-phase flow of a fluid infused with particles. Int. J. Non. Linear. Mech. 59, 76–82 (2014).

[b59] Wu W.-T., Aubry N., Massoudi M., Kim J. & Antaki J. F. A numerical study of blood flow using mixture theory. Int. J. Eng. Sci. 76, 56–72 (2014). 24791016 10.1016/j.ijengsci.2013.12.001 PMC4002018

[b60] Fabre J.-E. . Decreased platelet aggregation, increased bleeding time and resistance to thromboembolism in P2Y1-deficient mice. Nat. Med. 5, 1199–1202 (1999). 10502826 10.1038/13522

[b61] Savage B., Cattaneo M. & Ruggeri Z. M. Mechanisms of platelet aggregation. Curr. Opin. Hematol. 8, 270–276 (2001). 11604561 10.1097/00062752-200109000-00002

[b62] Purvis J. E., Chatterjee M. S., Brass L. F. & Diamond S. L. A molecular signaling model of platelet phosphoinositide and calcium regulation during homeostasis and P2Y1 activation. Blood 112, 4069–4079 (2008). 18596227 10.1182/blood-2008-05-157883 PMC2581995

[b63] Monroe D. M., Hoffman M. & Roberts H. R. Platelets and thrombin generation. Arterioscler. Thromb. Vasc. Biol. 22, 1381–1389 (2002). 12231555 10.1161/01.atv.0000031340.68494.34

[b64] Kahn M. L., Nakanishi-Matsui M., Shapiro M. J., Ishihara H. & Coughlin S. R. Protease-activated receptors 1 and 4 mediate activation of human platelets by thrombin. J. Clin. Invest. 103, 879–887 (1999). 10079109 10.1172/JCI6042 PMC408153

[b65] Offermanns S. The role of heterotrimeric G proteins in platelet activation. Biol. Chem. 381, 389–396 (2000). 10937869 10.1515/BC.2000.051

[b66] Klages B., Brandt U., Simon M. I., Schultz G. & Offermanns S. Activation of G12/G13 results in shape change and Rho/Rho-kinase–mediated myosin light chain phosphorylation in mouse platelets. J. Cell Biol. 144, 745–754 (1999). 10037795 10.1083/jcb.144.4.745 PMC2132941

[b67] OpenCFD. OpenFOAM Programmer’s Guide Version 2.1.0 (2011).

[b68] Henderson A., Ahrens J. & Law C. The ParaView Guide (Kitware Clifton Park, NY, 2004).

[b69] Frojmovic M. M., Mooney R. F. & Wong T. Dynamics of platelet glycoprotein IIb-IIIa receptor expression and fibrinogen binding. I. Quantal activation of platelet subpopulations varies with adenosine diphosphate concentration. Biophys J 67, 2060 (1994). 7858143 10.1016/S0006-3495(94)80689-7 PMC1225581

[b70] Richardson P. D. Effect of blood flow velocity on growth rate of platelet thrombi. Nature 245, 103–104 (1973). 4582758 10.1038/245103a0

[b71] Hellums J. D. 1993 Whitaker Lecture: biorheology in thrombosis research. Ann. Biomed. Eng. 22, 445–455 (1994). 7825747 10.1007/BF02367081

[b72] Engelberg H. & Dudley A. Plasma heparin levels in normal man. Circulation 23, 578–581 (1961). 13696820 10.1161/01.cir.23.4.578

[b73] Aoki H., Mizobe T., Nozuchi S. & Hiramatsu N. *In vivo* and *in vitro* studies of the inhibitory effect of propofol on human platelet aggregation. J. Am. Soc. Anesthesiol. 88, 362–370 (1998). 10.1097/00000542-199802000-00015 9477057

[b74] Neeves K. B. & Diamond S. L. A membrane-based microfluidic device for controlling the flux of platelet agonists into flowing blood. Lab Chip 8, 701–709 (2008). 18432339 10.1039/b717824g PMC2612095

[b75] Fogelson A. L. & Guy R. D. Platelet–wall interactions in continuum models of platelet thrombosis: formulation and numerical solution. Math. Med. Biol. 21, 293–334 (2004). 15567887 10.1093/imammb21.4.293

[b76] Feng D. . Platelet glycoprotein iiia pla polymorphism, fibrinogen, and platelet aggregability the framingham heart study. Circulation 104, 140–144 (2001). 11447076 10.1161/01.cir.104.2.140

[b77] Weiss H. J. Platelets: pathophysiology and antiplatelet drug therapy (AR Liss, 1982).

[b78] Martinuzzo M. E., Maclouf J., Carreras L. O. & Levy-Toledano S. Antiphospholipid antibodies enhance thrombin-induced platelet activation and thromboxane formation. Thromb. Haemost. 70, 667–671 (1993). 8115993

[b79] Nunn D. L. & Watson S. P. A diacylglycerol kinase inhibitor, R59022, potentiates secretion by and aggregation of thrombin-stimulated human platelets. Biochem. J. 243, 809–813 (1987). 2821994 10.1042/bj2430809 PMC1147929

[b80] Jones R. L., Wilson N. H. & Marr C. G. In: Chemistry, Biochemistry, and Pharmacological Activity of Prostanoids (Oxford: Pergamon Press, 1979).

[b81] Remuzzi G. . Reduced platelet thromboxane formation in uremia. Evidence for a functional cyclooxygenase defect. J. Clin. Invest. 71, 762 (1983). 6298281 10.1172/JCI110824 PMC436927

[b82] Von Holst H., Granström E., Hammarström S., Samuelsson B. & Steiner L. Effect of leucotrienes C4, D4, prostacyclin and thromboxane A2 on isolated human cerebral arteries. Acta Neurochir. (Wien). 62, 177–185 (1982). 7048861 10.1007/BF01403622

