# Supplementary material for: Multi-Constituent Simulation of Thrombus Deposition
Source: Sci Rep. 2017 Feb 20;7:42720. doi: 10.1038/srep42720 (PMC5316946; doi:10.1038/srep42720)
Supplement: Supplementary Information [file srep42720-s1.pdf]

Multi-Constituent Simulation of Thrombus Deposition

Wei-Tao Wu[1], Megan A. Jamiolkowski[2.3], William R. Wagner[2,3,4,5], Nadine Aubry[6], Mehrdad Massoudi[7], James F. Antaki[1*]

1. Department of Biomedical Engineering, Carnegie Mellon University, Pittsburgh, PA, 15213, USA
2. McGowan Institute for Regenerative Medicine, Pittsburgh, PA, USA
3. Department of Bioengineering, University of Pittsburgh, Pittsburgh, PA, USA
4. Department of Surgery, University of Pittsburgh, Pittsburgh, PA, USA
5. Department of Chemical Engineering, University of Pittsburgh, Pittsburgh, PA, USA
6. Department of Mechanical Engineering, Northeastern University, Boston, MA, 02115, USA
7. U. S. Department of Energy, National Energy Technology Laboratory (NETL), PA, 15236, USA

* antaki@cmu.edu

Appendix 1. Governing questions of chemical/biological species

Provided below are the full set of governing equations in expanded form. This model is an extension of our previous model (Sorensen et al. [1,2]). All the changes are denoted in **boldface**.

Unactivated resting PLTs in flow ([RP])

$$\frac{\partial[RP]}{\partial t} + div\left(\boldsymbol{v}_f \cdot [RP]\right) = div(D_P \cdot \nabla[RP]) - k_{apa}[RP] - \boldsymbol{k_{spa}}[\boldsymbol{RP}] - \boldsymbol{k_{rpd}}[\boldsymbol{RP}] \tag{1}$$

where $\boldsymbol{v}_f$ is the velocity of the fluid (blood), D_P is the diffusivity of the platelets, **$\boldsymbol{k_{apa}}$ and $\boldsymbol{k_{spa}}$ are the platelets activation rate due to agonists and shear stress, and $\boldsymbol{k_{rpd}}$ is the deposition rate between [RP] and deposited platelets. The detail definition of the $\boldsymbol{k_{rpd}}$ in mathematical and numerical see Appendix 2.**

Activated platelets in flow [AP]

$$\frac{\partial[AP]}{\partial t} + div\left(\boldsymbol{v}_f \cdot [AP]\right) = div(D_P \cdot \nabla[AP]) + k_{apa}[RP] + \boldsymbol{k_{spa}}[\boldsymbol{RP}] - \boldsymbol{k_{apd}}[\boldsymbol{AP}] \tag{2}$$

where $\boldsymbol{k_{apd}}$ is the deposition rate between [AP] and deposited platelets. The detail definition of the $\boldsymbol{k_{apd}}$ in mathematical and numerical see Appendix 2.

Deposited resting platelets [RP$_d$]

$$\frac{\partial[\boldsymbol{RP_d}]}{\partial \boldsymbol{t}} = (\mathbf{1}-\boldsymbol{\theta})\boldsymbol{k_{rpd}}[\boldsymbol{RP}] - \boldsymbol{k_{apa}}[\boldsymbol{RP_d}] - \boldsymbol{k_{spa}}[\boldsymbol{RP_d}] - \boldsymbol{f_{emb}}[\boldsymbol{RP_d}] \tag{3}$$

where $(\mathbf{1}-\boldsymbol{\theta})\boldsymbol{k_{rpd}}[\boldsymbol{RP}]$ is the percentage of [RP] activated by contact with biomaterial surfaces and formatted thrombus, $\boldsymbol{f_{emb}}$ is the cleaning rate of deposited platelets due to shear stress.

Deposited activated platelets [AP_d]

$$\frac{\partial[\boldsymbol{AP_d}]}{\partial \boldsymbol{t}} = \boldsymbol{\theta k_{rpd}}[\boldsymbol{RP}] + \boldsymbol{k_{apd}}[\boldsymbol{AP}] + \boldsymbol{k_{apa}}[\boldsymbol{RP_d}] + \boldsymbol{k_{spa}}[\boldsymbol{RP_d}] - (\boldsymbol{f_{emb}} + \boldsymbol{f_{stb}})[\boldsymbol{AP_d}] \tag{4}$$

where $\boldsymbol{f_{stb}}$ is the stabilization rate parameter of [$\mathrm{AP_d}$], which convert deposited thrombus to be stabilized thrombus which is harder to be cleaned by shear stress.

Deposited and stabilized platelets [AP_s]

$$\frac{\partial[\boldsymbol{AP_s}]}{\partial \boldsymbol{t}} = \boldsymbol{f_{stb}}[\boldsymbol{AP_d}] \tag{5}$$

PLT-released agonists (ADP) [a_{pr}]

$$\frac{\partial[\mathrm{a_{pr}}]}{\partial t} + div(\boldsymbol{v}_f \cdot [\mathrm{a_{pr}}]) = div(D_{apr} \cdot \nabla[\mathrm{a_{pr}}]) + \lambda_j(k_{apa}[RP] + \boldsymbol{k_{spa}}[\boldsymbol{RP}] + \boldsymbol{k_{apa}}[\boldsymbol{RP_d}] + \boldsymbol{k_{spa}}[\boldsymbol{RP_d}] + \boldsymbol{\theta k_{rpd}}[\boldsymbol{RP}]) - k_{1,j}[\mathrm{a_{pr}}] \tag{6}$$

where λ_j is the amount of agonist *j* released per platelet and $k_{1,j}$ is the inhibition rate constant of agonist *j*; and *j* represents ADP for current specie. **Therefore $\boldsymbol{\lambda_j k_{apa}}[\boldsymbol{RP}]$ and $\boldsymbol{\lambda_j k_{apa}}[\boldsymbol{RP_d}]$ are the ADP releasing attributed to the activation of unactivated platelets due to agonist, $\boldsymbol{\lambda_j k_{spa}}[\boldsymbol{RP}]$ and $\boldsymbol{\lambda_j k_{spa}}[\boldsymbol{RP_d}]$ are the ADP releasing attributed to the activation of unactivated platelets due to shear stress and $\boldsymbol{\lambda_j \theta k_{rpd}}[\boldsymbol{RP}]$ represents the ADP releasing attributed to the platelets activation due to contact. $\boldsymbol{k_{1,j}}[\mathbf{a_{pr}}]$ is the inhibition rate of ADP.**

PLT-synthesized agonists (TxA_2) [a_{ps}]

$$\frac{\partial[\mathrm{a_{ps}}]}{\partial t} + div(\boldsymbol{v}_f \cdot [\mathrm{a_{ps}}]) = div(D_{aps} \cdot \nabla[\mathrm{a_{ps}}]) + s_{pj}([AP] + [\boldsymbol{AP_d}]) - k_{1,j} \cdot [\mathrm{a_{ps}}] \tag{7}$$

where s_{pj} is the rate constant of synthesis of the agonist *j*; and *j* represents TxA_2 for current specie. **Therefore $\boldsymbol{s_{pj}}[\boldsymbol{AP}]$ and $\boldsymbol{s_{pj}}[\boldsymbol{AP_d}]$ represent the rate of synthesis of TxA_2 due to activated platelets in flow and deposited activated platelets.** $k_{1,j} \cdot [\mathrm{a_{ps}}]$ is the inhibition rate of TxA_2.

Prothrombin [PT]

$$\frac{\partial[\mathrm{PT}]}{\partial t} + div(\boldsymbol{v}_f \cdot [\mathrm{PT}]) = div(D_{PT} \cdot \nabla[\mathrm{PT}]) - \varepsilon[\mathrm{PT}](\phi_{at}([AP] + [AP_d]) + \phi_{rt}([RP] + [RP_d])) \quad (8)$$

where ε is the unit conversion which is from NIH units to SI units, ϕ_{at} and ϕ_{rt} are the thrombin generation rate constant on the surface of activated platelets and unactivated resting platelets. Therefore $\phi_{at}[AP]$ and $\phi_{rt}[RP]$ are the thrombin generation rate due to activated and resting platelets in flow, while **$\boldsymbol{\phi_{at}[AP_d]}$ and $\boldsymbol{\phi_{rt}[RP_d]}$ are the thrombin generation rate due to deposited activated and unactivated platelets.**

Thrombin [TB]

$$\frac{\partial[TB]}{\partial t} + div(\boldsymbol{v}_f \cdot [TB]) = div(D_{TB} \cdot \nabla[TB]) + [\mathrm{PT}](\phi_{at}([AP] + [AP_d]) + \phi_{rt}([RP] + [RP_d])) - \Gamma \cdot [TB] \quad (9)$$

where Γ is the Griffith's template model for the kinetics of the heparin-catalyzed inactivation of thrombin by ATIII. Therefore $\Gamma \cdot [TB]$ is the inactivation rate of thrombin by ATIII.

ATIII [AT]

$$\frac{\partial[AT]}{\partial t} + div(\boldsymbol{v}_f \cdot [AT]) = div(D_{AT} \cdot \nabla[AT]) - \Gamma \cdot \varepsilon[TB] \quad (10)$$

where $\Gamma \cdot \varepsilon[TB]$ is the consumption rate of ATIII due to inactivation of thrombin.

Appendix 2. Mathematical and Numerical Considerations for Deposition Rates k_{rpd} and k_{apd}.

Provided below are mathematical and numerical considerations about the two terms in equations (1) – (4) above, that represent the rate of deposition to the surface of the thrombus by unactivated resting platelets ($\boldsymbol{k_{rpd}}$) and activated platelets ($\boldsymbol{k_{apd}}$) in the free stream. Referring to the finite volume schematic depicted in Figure S1, the $\boldsymbol{k_{rpd}}$ of the finite-volume-cell 5 is calculated as

$$\boldsymbol{k_{rpd} = div(k_{pd,f}\vec{n})k_{ra}} \quad \mathbf{(11)}$$

where k_{ra} is a constant, $\vec{n}$ is the normal to the finite-volume-face, and f refers to the faces shared by adjacent cells 2, 4, 6 and 8. Assuming that thrombus tends to grow layer by layer, when the volume fraction of the deposited platelets of a finite-volume cell $\boldsymbol{\phi}$ is greater than a critical value $\boldsymbol{\phi_c}$ (for example, 0.74, which is the maximum packing fraction for spheres), this cell is able to influence the neighbor cells. Therefore

$$k_{pd,f} = \begin{cases} \frac{[AP_d]_f}{PLT_{max}}, \phi > \phi_c \\ 0, \phi < \phi_c \end{cases} \quad (12)$$

where $\frac{[AP_d]_f}{PLT_{max}}$ represents the percentage of the area occupied by deposited activated platelets at that mesh face. This rule does not apply to the boundary faces.

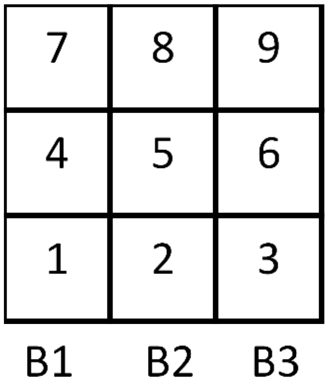

Figure S1 Schematic of cells and faces of a mesh.

References

1. Sorensen, E. N., Burgreen, G. W., Wagner, W. R. & Antaki, J. F. Computational simulation of platelet deposition and activation: I. Model development and properties. *Ann Biomed Eng* **27,** 436–448 (1999).

2. Sorensen, E. N., Burgreen, G. W., Wagner, W. R. & Antaki, J. F. Computational simulation of platelet deposition and activation: II. Results for Poiseuille flow over collagen. *Ann Biomed Eng* **27,** 449–458 (1999).
